# Nanoemulsions as novel nanocarrieres for drug delivery across the skin: In-vitro, in-vivo evaluation of miconazole nanoemulsions for treatment of *Candidiasis albicans*

**DOI:** 10.1080/15685551.2021.1965724

**Published:** 2021-08-17

**Authors:** Umar Farooq, Akhtar Rasul, Muhammad Zafarullah, Ghulam Abbas, Maria Rasool, Farman Ali, Shabbir Ahmed, Zeeshan Javaid, Zoya Abid, Humayun Riaz, Rana Khalid Mahmood Arshad, Shayan Maryam, Naseem Amna, Kanwal Asif

**Affiliations:** a. Department of Pharmaceutics, Faculty of Pharmaceutical Sciences, Government College University Faisalabad, Faisalabad, Punjab, Pakistan; b. Department of Pharmacy, Rashid Latif College of Pharmacy, Lahore, Pakistan; c. Emergency Department, Punjab Institute of Cardiology, Lahore, Pakistan; d. Government College University Faisalabad, Pakistan; e. Beaumont Hospital Dearborn, Michigan, USA; f. Department of Chemical Engineering, Centre for Synthetic Biology, College of Chemical and Biological Engineering, Zhejiang University, Hangzhou, China; g. Department of Pharmacy, Mirpur University of Science and Technology, Mirpur, Azad Kashmir, Pakistan; h. Department of Pharmacy, Islam College of Pharmacy, Sialkot, Pakistan

**Keywords:** Nanoemulsion, gelatin, miconazole, topical delivery system

## Abstract

In the current research, attempt is made to fabricate a nanoemulsion (NE) containing an antifungal agent. The prepared formulation has been expected to enhance skin penetration. It is also studied for in vitro drug release and toxicity assessment. Spontaneous titration method was used for preparation of NE. Prepared NE were characterized for their charge, size, morphology, rheological behaviour, drug release profile, skin permeability. The drug permeation and skin irritation were investigated. The in vitro antifungal activity was inspected using the well agar diffusion method. Miconazole NE showed good penetration in the skin as compared to marketed products. SEM showed semispherical shapes of the droplets. Zeta potential and zeta sizer showed that size was in nano ranges having positive charge.

## Introduction

Skin have been the largest organ of body as it covers all the body and is the first protection barrier of skin against pathogens and other infectious toxins as well as against environmental conditions like heat, cold [1]. Skin diseases are currently emerging threats because millions of people are affected with numerous skin infections [2]. Skin infections arises due to various pathogenic organisms which includes bacteria and fungi mostly [3]. Skin infections and disease may impose life alarming situations when left untreated.

Transdermal delivery of drugs with conventional techniques employed a lot of problem including poor penetration through skin so to overcome this barrier different nanocarrier techniques have been employed for the manufacturing of nanoparticles of different properties for the treatment of skin diseases [4]. Nanocarriers that have been developed and successfully studied for their in vitro and in vivo drug release properties, these include solid lipid nanoparticles (SLN), nanospheres, nanocapsules, nanogels, nanoemulsions (NEs), ethosomes, microemulsions, liposomes, dendrimers, micelles and etc [5]. All these nanocarriers have ability to easily cross the skin membrane and they can easily deposit under skin [6]. Pharmacokinetic properties of nanocarriers have also been improved remarkably as compared to conventional dosage forms [7]. Certain permeation enhancement techniques can also be employed to increasing the drug absorption and penetration through the skin like Ionophoresis, sonophoresis and pro drug technique.

Miconazole is a broad spectrum anti-fungal agent which have been primarily employed for the treatment of superficial infection caused due to any fungus [8]. However, its use has been limited due to poor solubility and poor penetration ability. In the current research miconazole have been incorporated into NE in which solubility of miconazole have also been improved along with its penetration through the skin with the help of NE have also been increased [9]. Gelatin a natural polymer has been used in the preparation of NE along with varying quantities of lecithin in few formulations.

## Materials

**Figure 1.**
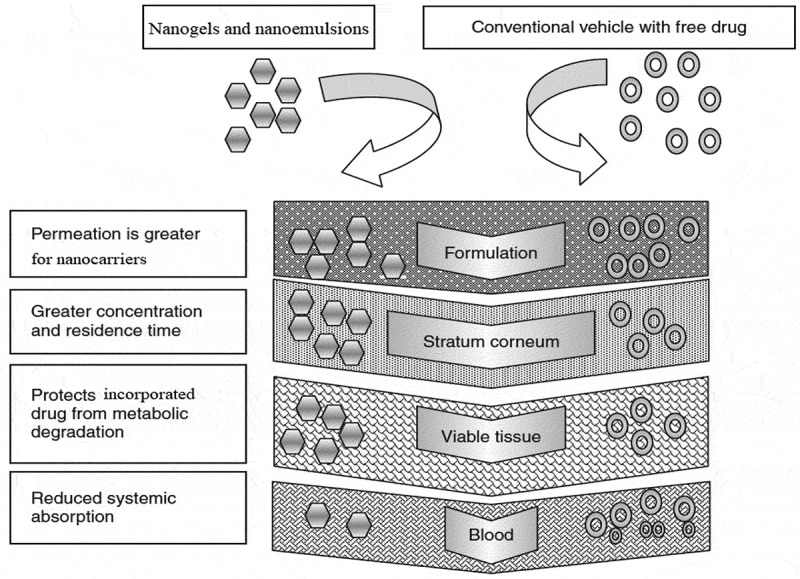
Comparison of penetration of nanocarriers and conventional dosage forms

**Figure 2.**
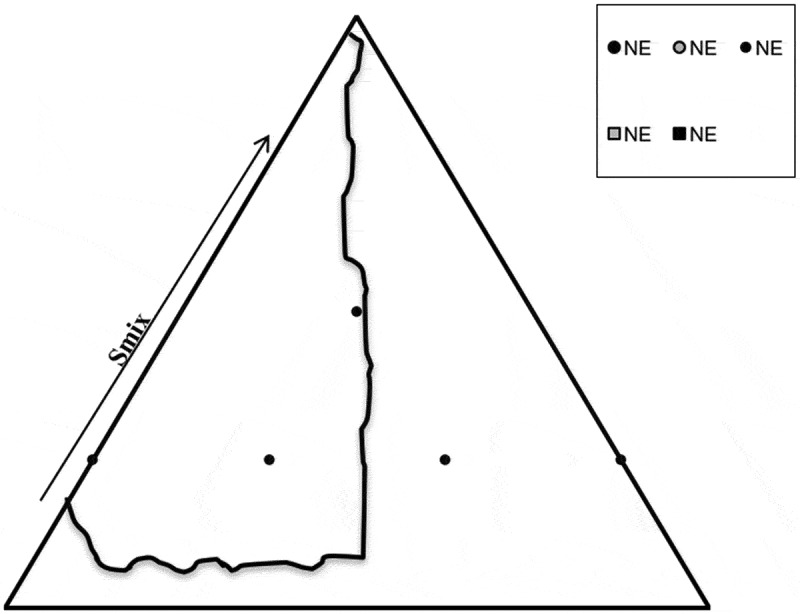
Ternary phase diagram

Poly (ethylene glycol) (PEG, Mn of 10,000 g/mol), Gelatin (GL) and Lecithin purchased from Musaji and Adam Chemicals (pvt.) Ltd. Pakistan. Miconazole was obtained as gift from Global Pharmaceuticals (pvt.) Ltd. Islamabad. Dichloromethane and ethanol were from purchased from Merck, USA. PEG 6000, acetone, acetonitrile were supplied from Musaji and Adam Chemicals (pvt.) Ltd. All chemicals and reagents were used as received without further purification unless stated otherwise Figure 1 and 2.

## Method of preparation

The stock solution of lecithin was prepared by dissolving the lecithin in water at concentration 3% w/v o. Gelatin stock solution was prepared by dissolving the gelatin in water at concentration 3% w/v. High pressure homogenization method was adopted for the preparation of NE. Miconazole was pre mixed in the ethanol solution, ethanol served as co-surfactant and then stock solution of gelatin and lecithin was incorporated into the miconazole ethanol solution. Briefly, prepared formulations were evaluated in terms of concentration of gelatin and lecithin [10]. Both had concentration in range of (0 to 3%) in aqueous phase. The amount of miconazole was 200 mg and ethanol which was used as co surfactant in oil phase had amount of (1 and 3 ml). Detailed formulations have been given in the table 4.1. At the end oil and aqueous phases were mixed to obtain a NE by using a T25 digital UlTRA TURRAX® homogenizer at 14,000 rpm and was stirred for about 5 minutes [11].

## Characterization of NE

### Pseudo-ternary phase diagram

Phase diagram was constructed by the water titration method and structure of NE that was formulated after emulsification was also identified. It also helped in characterizing the behaviour of NE along various dilution methods. The initial studies were conducted making mixtures for NEs contain the following ratios of 9:1, 4:1, 7:3, 3:2, 1:1, 2:3, 3:7, 1:4 for oil and surfactant. It was done at 25°C, kept for incubation for around 2 days of time i.e., 48 hours and observed afterwards. Then mixture of surfactant was incorporated into the oil to produce solutions of ratios 9:1, 4:1, 7:3, 3:2, 1:1, 2:3, 3:7, 1:4, 1:9 m/m. This mixture of varying ratios were kept in test tubes. Scanner was used to visualize turbidity and transparency of the phase diagram dilution line ratios. Mixtures of varying ratios were then characterized by zeta sizer for globular size analysis, zeta potential and PDI. Nanostructures of the prepared NEs were classified by electrical conductance method and it was found that if oil in water (o/w) or water in oil (w/o) had constituted as oil in water (o/w) NE which had high electrical conductance values as compared to water in oil (w/o) NE.

## Viscosity measurement

Viscosity is restrained to evaluate rheological properties of nano emulsion formulations. The viscosity NE formulations with and without miconazole was observed using Brookfield viscometer DV-II+ Pro (Brookfield Viscometers Ltd., UK) at 50 rpm with help of spindle 64 for 1 min. The procedure was completed at ambient temperature (25 ± 0.5°C) in triplicate (*n* = 3) and then average value was calculated [12].

## Conductivity measurements

Conductivity is determined to find the type of NE system i.e., oil/water or water/oil type. For this reason, conductivity meter (EcoScan con5, Eutech Instruments) was used to evaluate the conductivities of NE having no drug formulations and extract loaded NE formulations at 25 ± 0.5°C. Experiments were repeated in triplicate and average value was calculated [13].

## pH measurements

pH meter (HI 2210 Hanna, United States) was used to measure pH of NE formulations with and without miconazole at 25 ± 0.5°C. The values were taken by triplicate manner (*n* = 3) and then their average value was also calculated [13].

## Refractive index

To measure R.I of blank NE formulations and extract loaded NE formulations Abbe Refractometer (PCE instruments UK Ltd) was used. Sample of few drops of nano emulsion was placed on the slide and readings were noted. Measurements were taken at 25 ± 2°C in triplicate manner [14].

## Particle size and polydispersity index analysis

To determine globular size photon correlation spectrometer (Malvern Zetasizer, UK) was used. The NE sample was placed in a cuvette in a thermostatic chamber. Before taking the readings, the NE formulations were diluted with distilled water. The readings were taken three times at 25 ± 0.5°C [14].

## Zeta potential measurement

The zeta potential of NE without miconazole formulations and miconazole loaded NE formulations were also evaluated using Malvern Zeta sizer. Readings were taken in triplicate manner (*n* = 3). The samples of nano emulsion were aptly diluted with distilled water before taking measurements [15].

## Drug content determination

For drug content analysis of miconazole loaded NE formulations, 1 g of sample was taken and diluted with methanol. Then mixture was vortexed thoroughly and sonicated in ultrasonicator machine [16]. Then measurements were taken by HPLC by appropriate dilutions with methanol at respective *λmax, i.e., 250 nm. The readings were taken in triplicate (n = 3).*

## In-vitro release studies

The in-vitro drug release of NE formulations containing miconazole were examined using Franz diffusion cells. The cell of the said apparatus consists of donor compartment and a receptor compartment. The diffusion area of single cell of apparatus was 1.538 cm^2^. The volume of liquid in receptor compartment portion was 10 mL. The cellulose acetate membrane was hydrated for twenty-four (24) hours in basic buffer (phosphate buffer) before start of study. The medium used in the receptor compartment was basic buffer having 7.4 pH. The temperature of medium was maintained by circulating water bath at controlled temperature of 32.0 ± 0.1°C. Cells were placed on magnetic stirrer hot plate for continuous stirring of cell medium [16]. When the temperature was maintained, 1 gram of NE formulation was applied on cellulose acetate membrane (Micropore, USA) that was placed over donor compartment of cell having pore size 0.45 µm and diameter of 13 mm. 1 mL of sample was taken out from receptor compartment after fixed time breaks of 5 and 30 minutes, and then at 1, 2, 3, 4, 5, 6, 8, 12 and 24 hours. 1 mL of basic buffer, i.e., phosphate buffer having pH 7.4 was added into receptor compartment after each sampling break time to maintain the sink environment. The obtained samples were afterwards evaluated for miconazole content using HPLC at respective C18 column, 1 mL/min flow rate, defined mobile phase ratio and *λmax*. Three replicates were performed for each formulation [17].

## Ex-vivo release studies

Ex-vivo studies for drug release were made using rat abdominal skin as the membrane. Ex-vivo skin permeation studies were also passed out using franz diffusion cell [16].

## Preparation of skin for skin toxicity studies

Mice of weight 250–300 g were taken and abdominal skin samples were excised. The hairs were removed carefully by applying hair removing cream at the abdomen. After 15 minutes, hairs were removed gently and carefully. Hairs were removed on the day when release studies were to be carried out in order to get optimal results. The rats were anesthetized before sacrificing them. Skin was removed and epidermis layer was separated from dermis layer carefully using scalpel. Subcutaneous fats were also removed. Then the skin was rinsed and washed with distilled water [18].

To start the release studies, the excised skin was fastened between the donor and the receptor compartment of diffusion cell in such a way that epidermis or stratum corneum part should face the donor compartment. Same procedure was used as mentioned in in-vitro permeation studies of nano emulsion. The collective drug release per unit area calculated as (µg/cm2), steady state flux represented by (Jss), permeability coefficient and lag time were evaluated as described above.

## Estimation of In-vitro anti-fungal activity

The capacity to reduce the infection due to fungi which was ideal property of miconazole loaded NE formulation, was evaluated by protein denaturation study. The procedure was implemented from work of [19] with slight modifications. Different concentrations of NE novel preparation were organized so that final concentrations of 30.27, 60.4, 119, 210, 505, 750 mg/mL were prepared. 5 mL of reaction mixtures were prepared each comprising 0.2 mL of egg albumin obtained from (fresh hen’s egg), 2 mL of nano emulsion sample with different concentrations and 2.9 mL of basic buffer phosphate buffer (0.2 M, pH 7.4). Similar volume of egg albumin, phosphate buffer and distilled water was taken as control. The mixtures were placed in incubator (EHRET, Germany) at 37°C for 15 min. Then reaction mixtures were placed in water bath at 55°C for 15 minutes to persuade the denaturation. Then samples were cooled and absorbance was measured at 660 nm. miconazole drug solution with similar concentrations was used as reference drug. Similar procedure was adopted for reference drug reaction mixtures and absorbance was noted. The percentage (%) inhibition by the nano emulsion of protein denaturation was premeditated as follows:

1 $$Percentage\;inhibition=\frac{Control-Tread\;Sample}{Control}\times 100$$

## Stability studies

Stability of NE formulations containing miconazole was evaluated. 5 ml of each formulation was taken and added into glass vials and then sealed. These vials were kept at room temperature (35 ± 1°C) for 90 day almost 3 months. Then the samples were analyzed visually for clarity, phase separation and drug precipitation. They were also analyzed for miconazole content, viscosity, conductivity and pH values [20].

The NE formulations were also subjected to centrifugal stability studies. The samples were centrifuged in a centrifuge machine (HERMLE, Germany) at 6000 rpm for 30 min. Then the formulations were observed for precipitation of drug, phase separation and clarity. All experiments were repeated three times (*n* = 3).

## Skin sensitivity, toxicity and histological examination studies

The purpose of these studies was to detect the skin sensitizing and irritant potential of the optimized formulation after topical application. Female Swiss albino mice of 20–25 g were selected for these studies. The negative control group was treated with normal saline 45 while formalin was used as a standard irritant for the positive control group. The dorsal region of mice was shaved 24 h prior to the application of the formulations using a hair remover cream (Veet®, Reckitt Benchiser). Then, 200 mg of NE containing 2 mg miconazole was applied on the shaved area with uniform spreading once daily for three consecutive days (*n* = 3). The skin was checked for any visible difference, such as erythema after the application of formulation for succesive 3 days. The erythemal scores were recorded (ranging from 0 to 4) on the basis of the degree of redness as follows: no redness = 0; slight redness (barely perceptible-light pink) = 1; moderate redness (dark pink) = 2; moderate to severe redness (light red) = 3; and severe redness (extreme erythema) = 4 grade.

On the third day, animals were sacrificed, and the skin was cut and processed. Briefly, specimens from control groups and the test group were fixed in 10% (v/v) buffered formalin. Subsequently, each tissue was rinsed thoroughly with water, dehydrated using a graded series of alcohols, embedded in paraffin wax, and microtomed. Then, the sections were stained with hematoxylin and eosin followed by observation under a high-power light microscope.

## Fourier transforms infra-red spectroscopy (FT-IR)

FT-IR spectra of miconazole raw, gelatin and miconazole loaded NE were chronicled using FT-IR spectrometer. Drug, polymer and NE were scanned in the range of 4000 cm^−1^ to 400 cm^−1^ with 32 scans per sample.

## Statistical analysis

The mean and standard deviation of all the results were calculated. All the results calculated from solubility study, viscosity, conductivity, globular size, polydispersity index, zeta potential, stability studies and miconazole loaded NE formulations were treated statistically using one-way analysis of variance (ANOVA). The results between blank and miconazole loaded NE formulations were compared using student’s t-test. The results were statistically significant if *p* < 0.05 C and confidence interval was taken 95%.

## Evaluation of NE

### Construction of phase diagram

Oil and surfactant solution were taken in all ratio for the surfactant to obtain a clear solution for 40 minutes, then vortex mixing was done in which it was rotated at 800 rpm for approximately 45 sections to obtain turbid mixtures. The fifth dilution that was made and it resulted in to formulation of kinetically stabe and transparent solution. It was an isotrpic system which included the properties of Winsor IV type behaviour for up to 3 months at the 25°C. As the proportion of oil in the nanoamulsion was increased gradually, it resulted in the increase in the droplet size of the NE and droplet size was also increased when the quantity of gelatin was also increased. In the meanwhile when oil was increased the quantity of lecithin was also decreased which also resulted in increase of droplet size. When the ratio of surfactant and oil was changed to 3:1, a pseudo binary NE was obtained which had the smallest globular size as shown in results by zeta size and PDI from the all-other ratios used in NE formulation. It was observed that five different regions were formed, i.e, Winsor IV when transparent NE was formed and milky Winsor IV when cloudy isotropic NE was used. Translucent Winsor I, II and III, milky Winsor I, II and III in addition to a gel/semisolid region was formulated. In the formulation F05 which had the ratio 5 was able to form a clear transparent and isotropic NE which have water content ranging in 40 % v/v for the surfactant mixture. As we increased the amount of ethanol which is oil phase it resulted in decrease in the region area of NE. It also helped in formulating the two or three phase oil in water (o/w) NE region which had an increased area and it appeared as milky isotropic. Gel region area was decreased in size as the total amount of ethanol was increased in the NE. Interfacial film which was formed was flexible which suggested that solid structures were dislocated and fluid phase areas were increased in measurement.

## Formulation studies

Skin irritation of many cosmeceuticals have been observed to be decreased due to the presence of non-ionic surfactants. The surfactants whose HLB values are 10.0 or above 10.0 usually produces more stable emulsion as it can be inferred from pseudo phase ternary diagram 2. The stable emulsion is formed with the incorporation of lecithin which act as surfactant in the formulation.

## Scanning electron microscopy (SEM)

**Figure 3.**
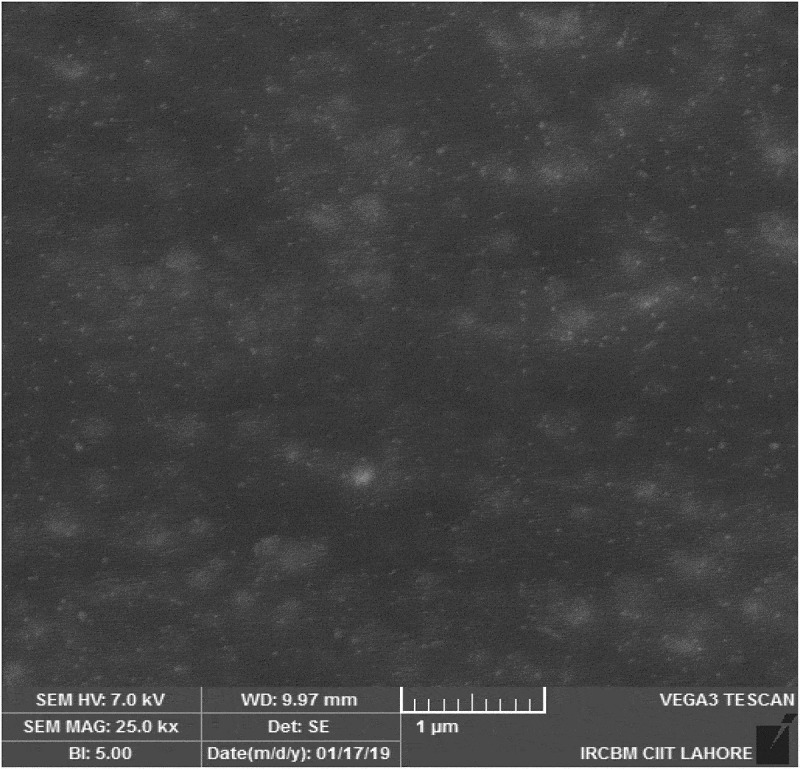
(a): SEM image of miconazole loaded nanoemulsions at 1 µm. (b): SEM image of miconazole loaded nanoemulsions at 500 nm. (c): SEM image of miconazole loaded nanoemulsions at 200 nm

**Figure 4.**
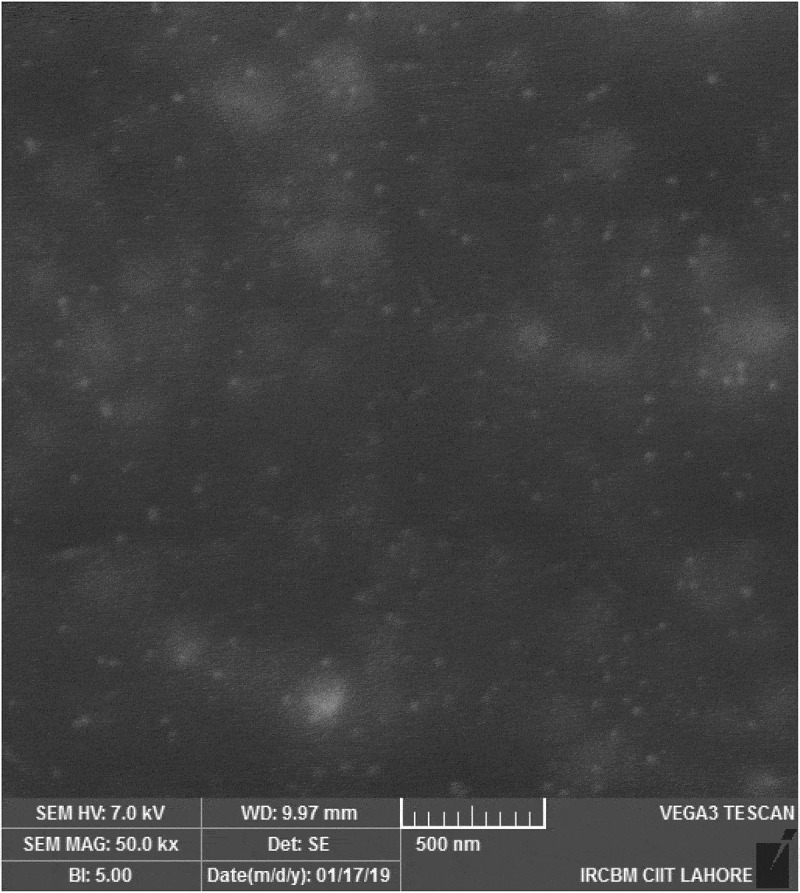
Size range of blank nanoemulsion(blank)

Surface morphology and shape of the prepared NEs was observed with the help of scanning electron microscopy. As image 3 (a,b and c) reveal that prepared NEs are moderately spherical and oval in shape. The SEM images taken at different resolutions i.e. having scale of 200 nm, 500 nm and 1 µm. The distrubution of two phases i.e. NE and continous phase smooth somewhat. Particle size range is also in nanometers as depicted by SEM. Some of the prepared NEs were observed in the form of clusters and mostly uniform dispersion of the miconazole all over the NEs have been shown in Figure 3 (a,b and c) by SEM at 1µm, 500 nm and 200 nm respectively Figure 4 and 7.

**Figure 5.**
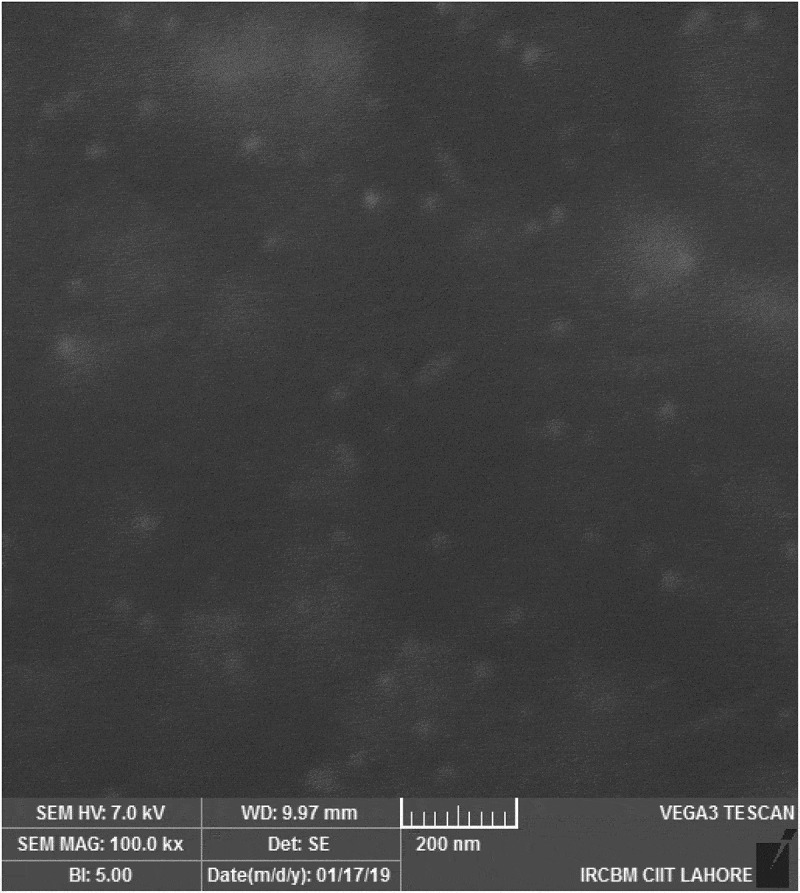
Size range of nanoemulsion F5 loaded with miconazole

**Figure 6.**
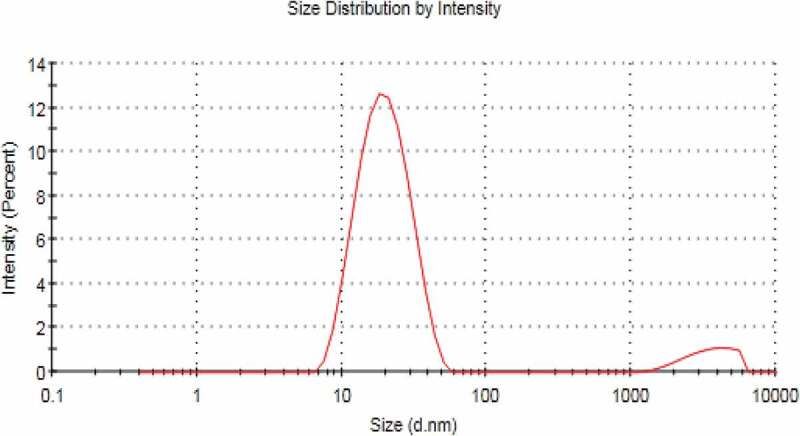
Zeta potential of blank nanoemulsion formulation

**Figure 7.**
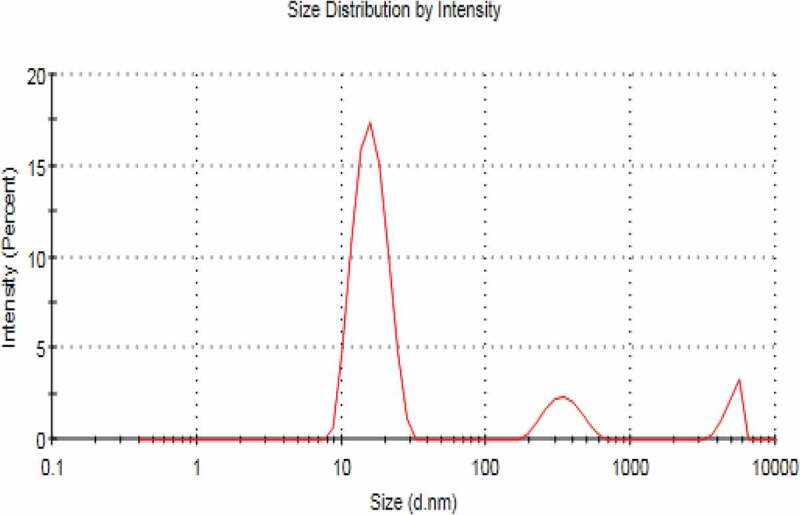
Zeta potential of miconazole loaded nanoemulsion formulation

## Zeta sizer, zeta potential and PDI

NEs prepared have the size in nm ranges. The size ranges of prepared NEs as observed by the help of zeta sizer is 207 nm to 2601 nm suggesting that there is different particle size distribution in whole the formulation, the poly dispersibility index has been seen in the result 0.337.

## Viscosity measurements

**Figure 8.**
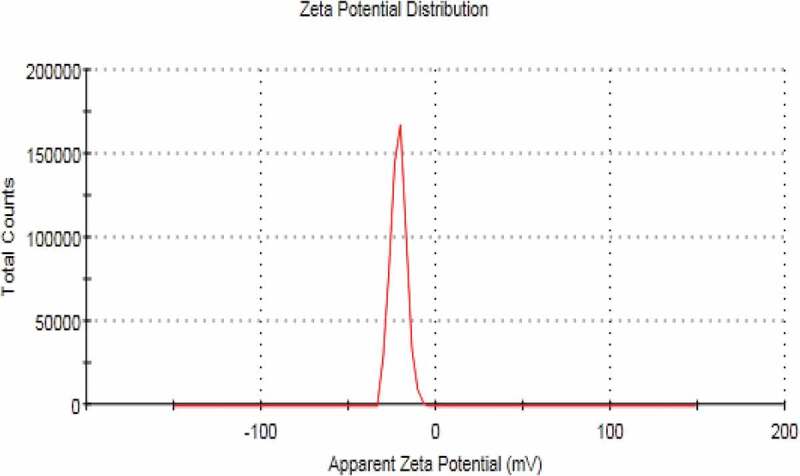
Graphic representation of viscosity of five Blank and miconazole loaded NE formulations

From Figure 8, it has been shown that there is a significant increase in viscosity as the amount of gelatin increased from 4% to 20%, Smix of lecithin and ethanol increased and water quantity decreased from 56% to 25% from formulations F1 to F5. The mean viscosities of blank NE formulations ranged from 24–493.93 cP and miconazole loaded NE formulations showed viscosity range from 26 to 505.27 cP. This shows that the viscosity increment in formulations was due to increment in the internal phase ratio in o/w NE. As the water content decreases the viscosity of formulation increases [21,22]. It is stated in some studies that, the viscosity of NE may be due to oil phase properties and droplet diameter of internal phase. In diluted nE formulations, the small number of droplets had no significant effect on viscosity of formulation, but when the number of droplets increase, droplet-droplet interactions increases and cause increase in friction which may lead to increase in viscosity [23]. Tween 80 has hydrophilic nature (HLB = 15) and its structure has large number of polyoxyethylene groups which tend to absorb the aqueous phase. This results in increase of viscosity due to reduction of free water of the formulations [24]. The results of viscosity among the blank and miconazole loaded NE formulations were considered significantly different (*p* < 0.05) from F1 to F5. Loading of NE cause slight increment in the viscosity of the NE formulations but the viscosity values between blank and miconazole loaded NE formulations were found to be not different significantly (*p* > 0.05).

## Conductivity measurement

**Figure 9.**
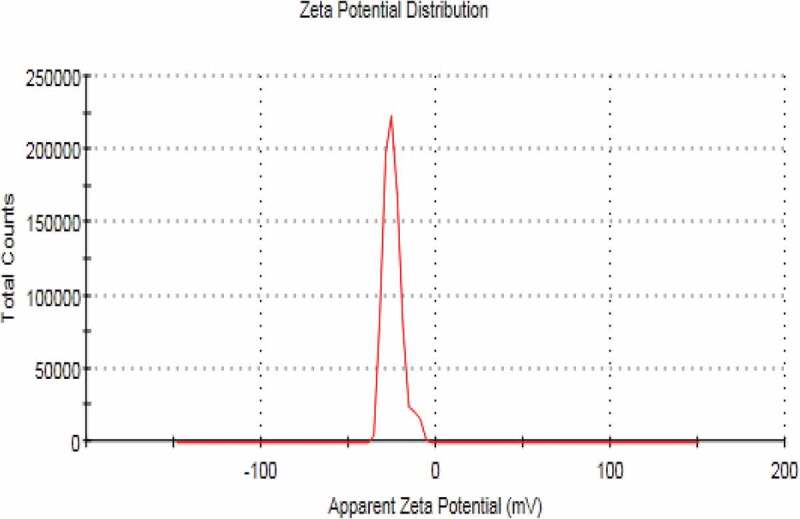
Graphic representation of conductivity values of five Blank and miconazole loaded NE formulations

The ability of aqueous phase to pass electrical current is known as conductivity. Electrical conductivity was measured to examine the type of microemulsion system. The electrical conductivity is different among o/w, w/o and bicontinous nE formulations. The conductivity is usually similar to normal water phase in o/w, very low in w/o and significantly high in bicontinous systems [25]. This conductivity property is due to presence of external water phase in o/w type. It was reported in a study w/o NE conductivity value is below 10 µS/cm and o/w ME has conductivity range from 10 to above 100 µS/cm [26]. The conductivity study of NE showed, with the increase in aqueous phase there is increase in conductivity values [27–29]. The blank NE formulations showed mean conductivity values ranging from 195.33 to 43.47 µS/cm while the formulations containing miconazole have conductivity values ranging from 207.2–50.67 µS/cm as shown in Figure 9. The decrease in conductivity values as we move from F1 to F5 is due to decrease in water content. miconazole loaded NE formulations and blank formulations show conductivity values to be significantly higher from F1 to F5. But there was no difference significantly in the conductivity values between blank & miconazole containing NE formulations.

## pH measurements

**Figure 10.**
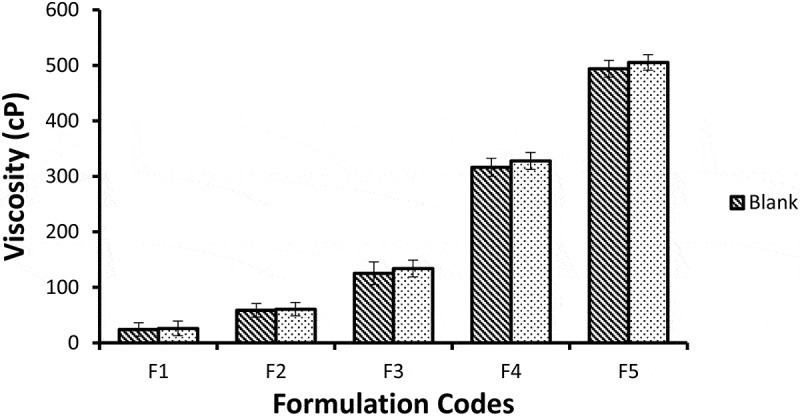
Graphic representation of pH values of five Blank and miconazole loaded NE formulations

The pH value of the skin ranges from 4.0 to 7.0 [30]. pH values of all blank and miconazole loaded NE formulations are within the acceptable range. Mean pH values of all blank formulations ranges from 4.99 ± 0.06 to 6.7 ± 0.1 while NE formulations containing miconazole have pH range from 5.47 ± 0.06–6.8 ± 0.06 shown in Figure 10. As the water content increases from F5 to F1 the pH value decreases. This may be due to low pH value of distilled water used (pH_used water_ = 5.45) [31]. The pH values were higher significantly (*p* < 0.05) among NE formulations with and without miconazole from F1 to F5. The results between blank NE formulations and formulations containing miconazole were not significantly different (*p* > 0.05).

## Refractive index

**Figure 11.**
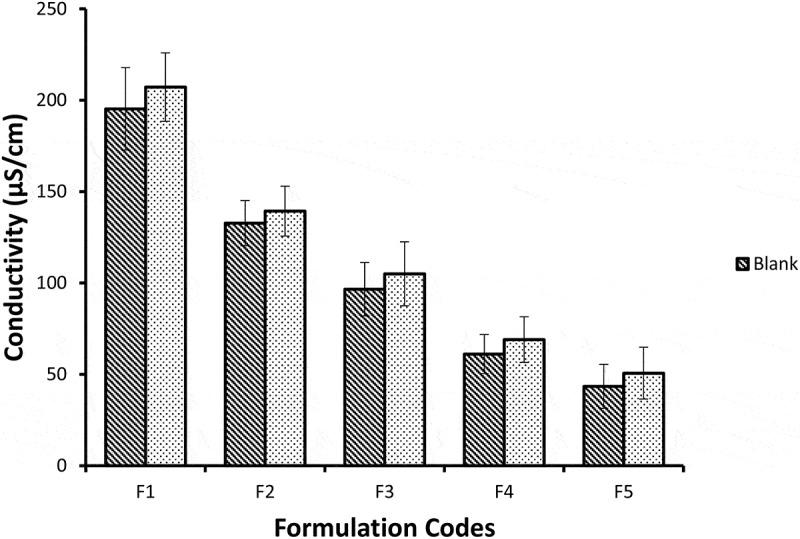
Graphic representation of refractive index values of five Blank and miconazole loaded NE formulations

Refrective index is represented by RI and is defined as the ratio of speed of light in vaccum to the speed of light in the particular substance or medium. As RI is the ratio between same value so it has no units. Refrective index (RI) is a physical property of substances and is usually used for the confirmation of substance nature, substance purity and concentration. The refractive index (RI) indicates the isotropy of the NEs. The mean values of refractive index of blank NE formulations ranged between 1.3829 ± 0.005 and 1.4289 ± 0.004. Miconazole loaded NE formulations showed mean refractive index values ranging from 1.3967 ± 0.003 to 1.4369 ± 0.003 shown in Figure 11. As water is the external phase in o/w NE, the R.I value is lower as compared to w/o NE because of lower R.I of water (1.3325). As the concentration of water phase decrease RI for the NE formulations also decreased. Refractive index increases from F1 to F5 as the oil phase and surfactant increases because of large refractive index value of ethanol (1.434) and lecithin (1.473). The R.I values were higher significantly (*p* < 0.05) among NE formulations with and without miconazole from F1 to F5. The results between blank NE formulations and formulations containing miconazole were not significantly different (*p* > 0.05).

## Drug content

**Figure 12.**
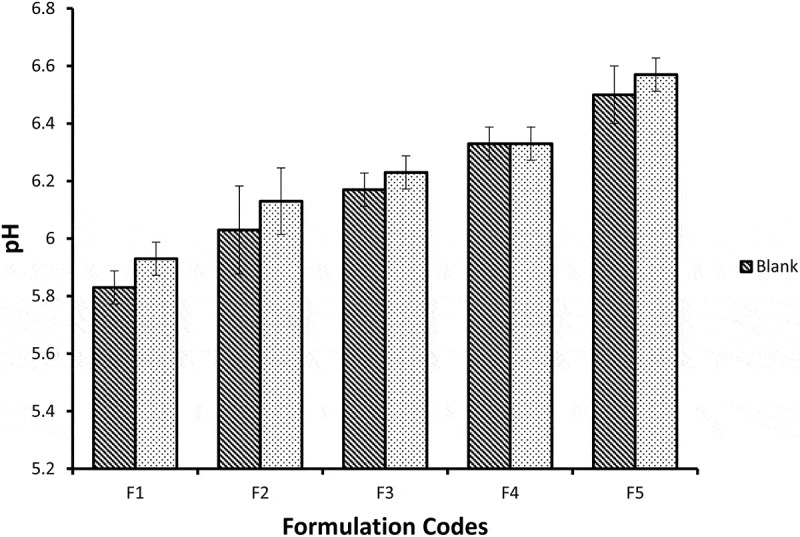
Graphic representation of % drug content values of five miconazole loaded NE formulations

Miconazole content of all NE formulations containing ranged from 98.6 ± 0.74 to 99.6 ± 0.79%. The obtained data revealed the uniform distribution of the drug within all studied NE formulations. The NE formulations were not different significantly (*p* > 0.05). The graphical representation is shown in Figure 12. All the results of viscosity, conductivity, R.I, drug content and pH are mentioned in table.

## Skin sensitivity, toxicity and histological examination studies

**Figure 13.**
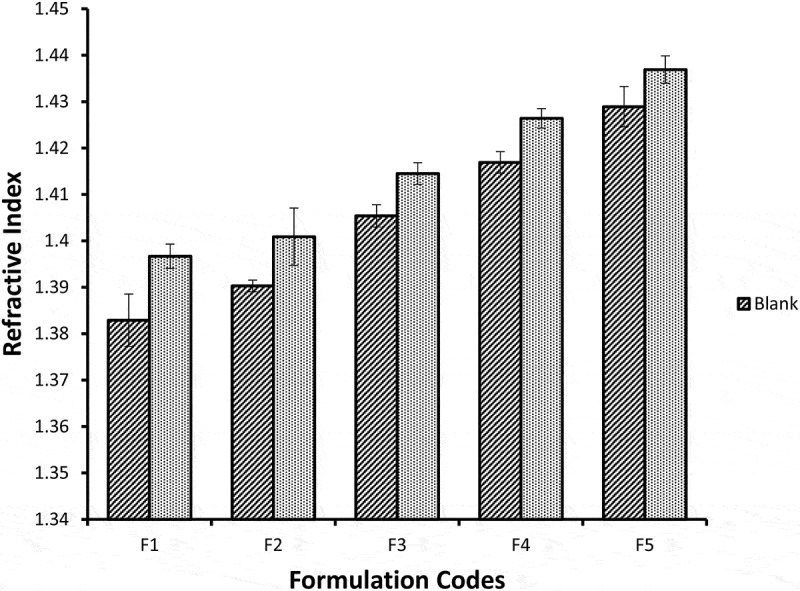
Vertical section histological microphotograph of mouse skin with the staining of hematoxylin and eosin A) formalin treated Positive control at view of lower power (×100); (B) formalin treated negative control at view of lower power (×100); (C) formalin treated Positive control at view of higher power (×400); (D)saline treated negative control at view of higher-power (×400)

**Figure 14.**
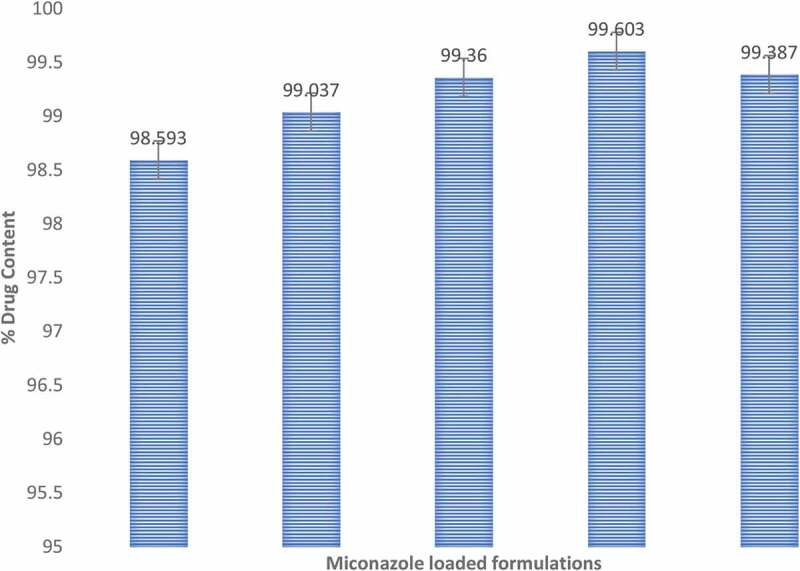
Vertical section histological microphotograph of mouse skin after staining with hematoxylin and eosin A) Placebo treated skin sample at view of lower power (×100); (B) miconazole loaded nanoemulsion (1%w/w) skin sample at view of lower power (×100); (C) saline treated skin sample at view of higher power (×400); (D) miconazole loaded nanoemulsion treated skin at view of higher-power (×400)

Topical formulation should be evaluated not only in terms of carrier capacity and enhancing drug permeation through the skin but also in terms of its tolerability and toxicity. NEs were composed of large amounts of surfactants and oil; hence, it was particularly important to consider the potential skin irritation and toxicological reactions resulting from topical application. The test was carried out for both blank and miconazole loaded NE. After application of the NE on the skin of mice it was observed for erythema scores after one day, two days and three days of time. Then the erythema was observed and it numbered from 0 to 4. No erythema was observed till the second day, i.e., till 48 hours after application of NEs. Hence the formulation prepared appeared to be safe for long term use but the conventional cream containing miconazole showed erythema of grade 2 after 3 days. Then the skin of mice was observed under the microscope after sacrificing the mice and preparaing the slides of the mice skin. FromFigure 13 it was observed that no sign of irritation and toxicity was shown on skin of mice after application of NE. NEs showed good penetration into the skin as well causing almost no irritation to the mice skin. Then the vertical section histological microphotograph of mouse skin after staining with hematoxylin and eosin was taken as shown in Figure 14 and it was once again confirmed that there was no sign of toxicity observed in the mice skin with NE but formalin and eosin as depicted Figure 13 produced erythema in the skin and local irritation on the mice skin was also observed as skin was tornout due to presence of formalin and eosin. Lecithin present in the NE formulation also facilitated the penetration of NE into the skin as lecithin had the ability to weaken the stratum corneum of skin which is first line skin barrier. When SC got weakened NE penetrated into the skin easily.

Hence it was concluded from histological monographs that NE containing gelatin and lecithin for delivery of miconazole into skin are safe and nonirritant for the skin. They are effective carrier system for the dermal delivery of drugs with no irritation potential.

## DSC and TGA

**Figure 15.**
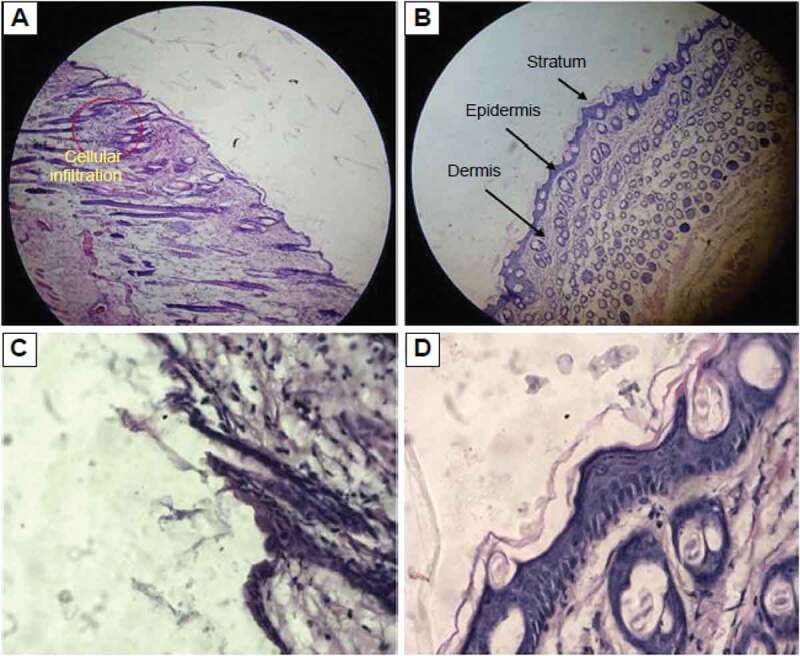
DSC of Naoemulison, gelatin and miconazole

**Figure 16.**
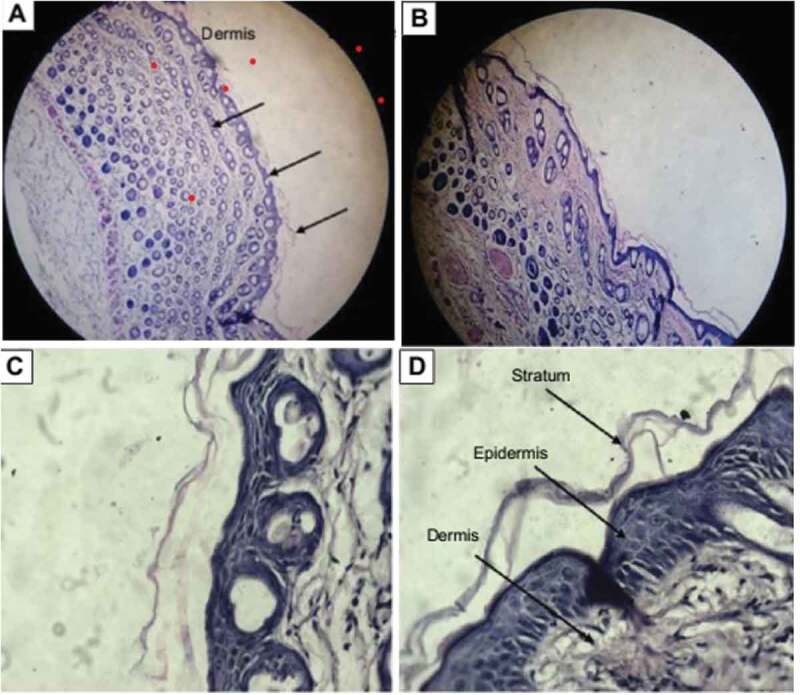
TGA of Naoemulison, gelatin and miconazole

DSC thermo grams of pure drug, gelatin, and drug loaded NE are presented in Figure 15. The thermo gram of DSC clearly indicates a sharp melting peak of miconazole at about 220.2 °C. The drug NE showed an absence of drug melting peak which indicates molecular dispersion of drug in the prepared NE. The gelatin showed melting peak near 500 °C while drug loaded NE did not show any endothermic transitions due to polymer network formation and molecular dispersion of the drug as depicted in Figure 16.

## X-ray diffraction studies (XRD)

**Figure 17.**
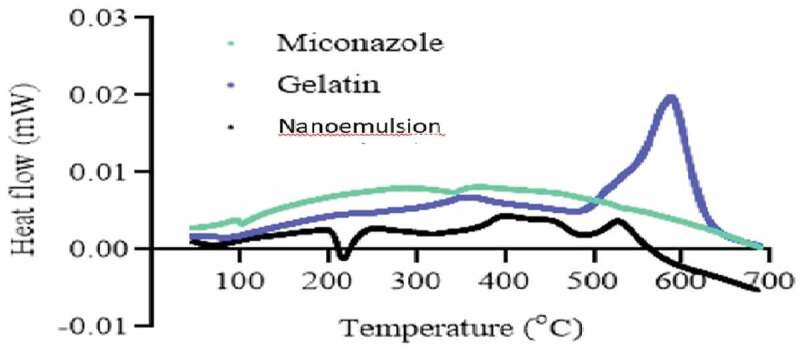
XRD of miconazole, gelatin and nanoemulsion

The XRD pattern of Gelatin, miconazole and miconazole loaded gelatin NE has been depicted in Figure 17. The diffract gram of unloaded miconazole loaded gel indicated peak at ~10.840°, 14.360°, 18.180°, 29.920°, 34.440° (2θ). While the diffract gram of miconazole indicated peaks at ~10.940°, 12.33°, 34.360°, 38.400°, 39.920°, 44.540°, 57.900° (2θ), while gelatin showed peaks at 10.40°, 24.60°, 28.100°, 29.20°, 34.40°, 47.00°. The results depict that the NE formation reduces the crystalline nature of both miconazole and gelatin. It can be concluded that NE formed is in amorphous form and contribute towards the enhanced solubility of the drug.

## Stability studies

**Figure 18.**
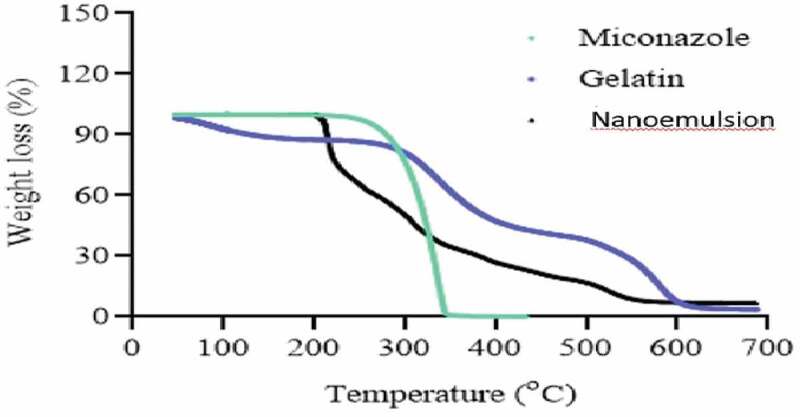
Graphical representation of stability data of viscosity study of all five NE formulations

**Figure 19.**
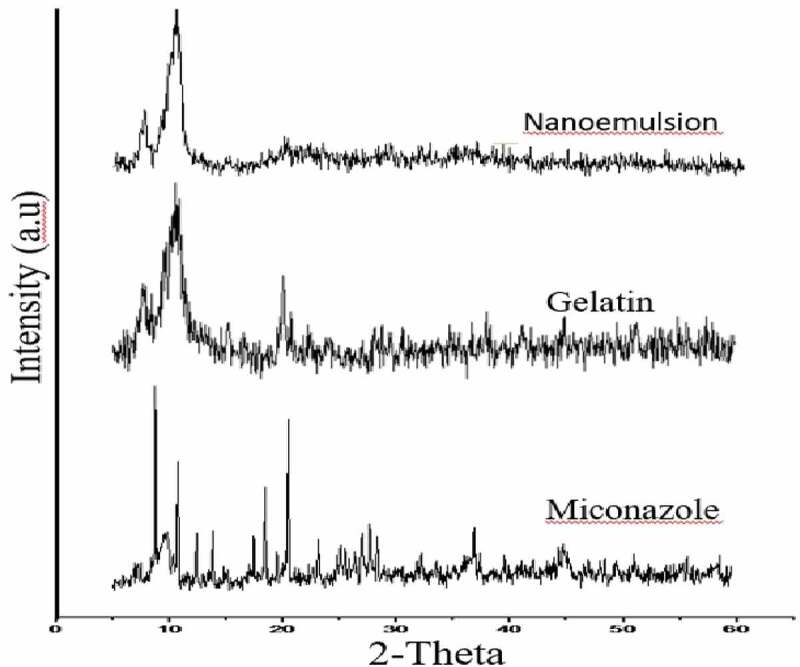
Graphical representation of stability data of conductivity study of all five NE formulations

**Figure 20.**
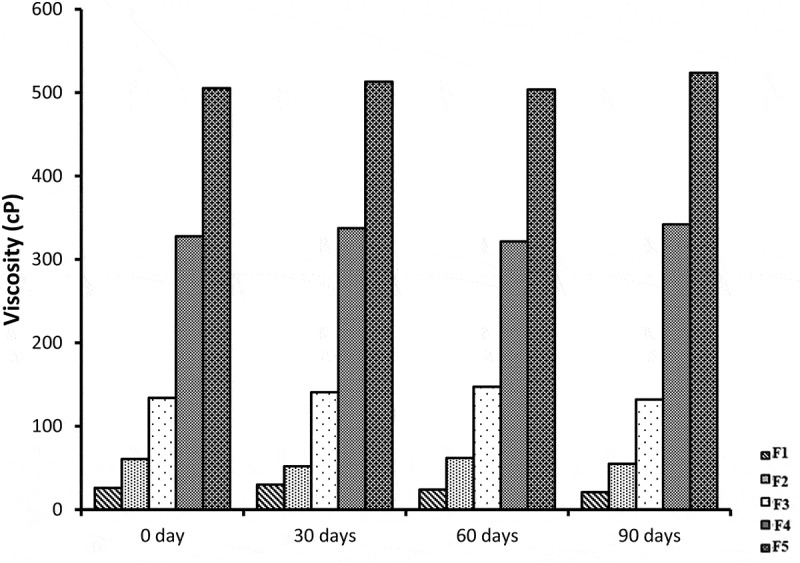
Graphical representation of stability data of % miconazole content of all five NE formulations

**Figure 21.**
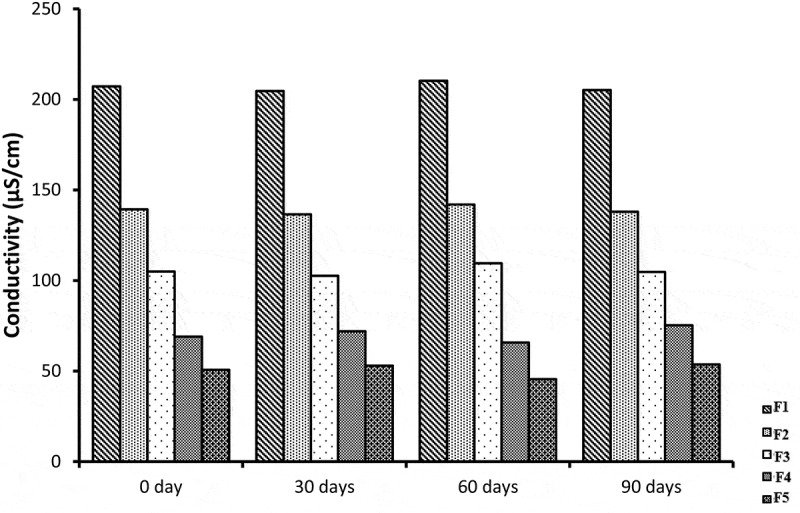
Graphical representation of stability data of pH measurements of all five NE formulations

All NE formulation loaded with miconazole were evaluated for stability studies. The visual appearance, pH values, viscosity, conductivity and % drug content were evaluated at 0 day, 30 days, 60 days and 90 days which showed almost similar results before and after storage at room temperature. The results of centrifugation test indicate that no precipitation of drug and no separation of phases occur. The results indicates that all NE formulations were stable physically and chemically. NE formulations before and after storage were not different significantly (*p* > 0.05) and shown in figures 18, 19, 20, 21.

## *In-vitro* release studies

**Figure 22.**
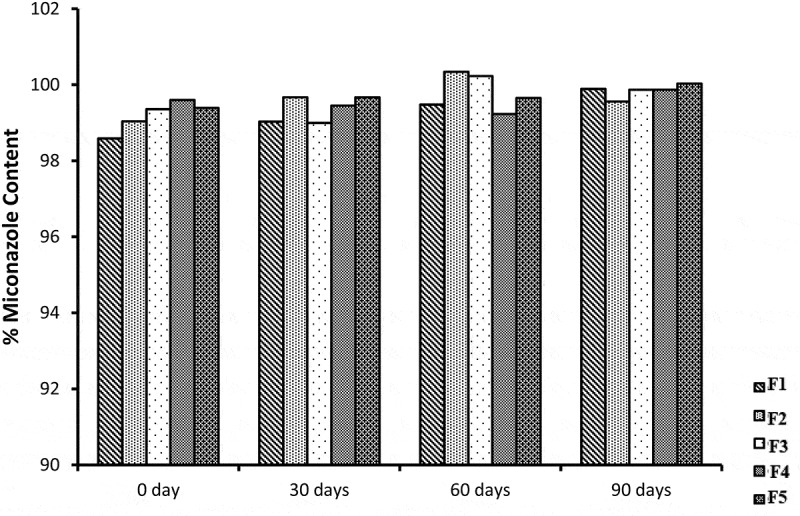
Permeation profiles of five NE formulations in comparison with control

The *in-vitro* release profiles of NE formulations containing miconazole were evaluated and cumulative drug release per unit time is demonstrated graphically in Figure 22. It was evaluated that maximum drug was release by formulation F01 and F05 showed lowest drug permeation. The order of release rate was F01> F03> F02> F04> F05> control. As the internal phase ratio is increased, there is gradual increase in viscosity of formulations due to which release rate was decreased [32,33]. Another possible reason is that, as the globular size increases the release rate decreases. This is because of the fact that the smaller the globular size, the more the formulation interacts with the skin as the surface area is increased. It was seen that drug release is decreased on increasing surfactant concentration. The possible reason is the increase in thermodynamic activity of the drug on increasing surfactant level. As thermodynamic activity is a driving force for the penetration of drug into the skin [34].

**Figure 23.**
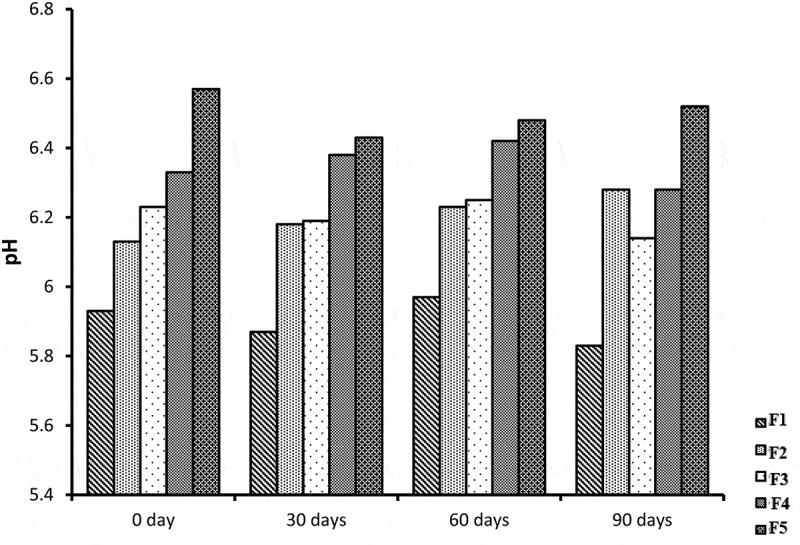
Permeation rate (flux) of five NE formulations in comparison with control. (*) represents significant difference of NE formulations flux from control

The permeation rate (flux) values and permeation coefficient values show significantly higher values (*p* < 0.05) as compared to control. This is because the formulations contain ethanol as oil which served as potent skin penetration enhancer and act by partitioning itself with lipid domains of stratum corneum [35,36]. Non-ionic surfactants like lecithin also served as strong penetration enhancers. NE formulations also contain lecithin and its glycol part which also acted as sorption enhancer. It acts as driving force for drug and takes it through skin by partitioning phenomena. Lag time values were also calculated. F01, F02, F03 showed greatest release and flux values and shortest lag time as compared to F04 and F05 due to low viscosity and small globular size. The results of flux, kP and lag time are given in Table 1 and 3. Flux of all formulations is shown graphically in Figure 23.

**Table 1.** Formulations of miconazole nanoemulsion

Sample	Aqueous Phase 10 ml		Oil Phase	
	Lecithin	Gelatin	Ethanol	Miconazole
F01	0 %	3 %	10 ml	200 mg
F02	1 %	2%	10 ml	200 mg
F03	2%	1%	10 ml	200 mg
F04	3%	0%	10 ml	200 mg
F05	1.5 %	1.5%	15 ml	200 mg

**Table 2.** Size ranges, PDI and zeta potential of Blank and miconazole loaded nanoemulsions

Formulation	Globular size (nm)		PDI		Zeta potential (mV)	
	Blank	Miconazole loaded nanoemulsion	Blank	Miconazole loaded nanoemulsion	Blank	Miconazole loaded nanoemulsion
F1	21.6 ± 2.39	22.33 ± 2.48	0.148 ± 0.004	0.157 ± 0.007	−22.67 ± 1.15	−22.77 ± 1.88
F2	34.97 ± 3.44	42.05 ± 3.95	0.246 ± 0.006	0.253 ± 0.006	−23.93 ± 0.76	−24.57 ± 0.21
F3	42.71 ± 3.30	48.13 ± 3.37	0.289 ± 0.004	0.287 ± 0.009	−24.43 ± 0.31	−24.83 ± 0.06
F4	61.20 ± 5.89	69.53 ± 6.82	0.305 ± 0.003	0.313 ± 0.006	−26.17 ± 0.35	−26.3 ± 0.36
F5	103.5 ± 4.25	112.4 ± 9.14	0.41 ± 0.006	0.421 ± 0.005	−28.7 ± 0.26	−29.1 ± 0.4

**Table 3.** In-vitro permeation parameters of NE formulations

Nanoemulsion codes	Flux (µg/cm^2^/hr)	Permeability coefficient (cm/hr)	Lag time (min)
F01	871.78 ± 6.43	0.109 ± 0.0008	1.25
F02	796.74 ± 10.84	0.099 ± 0.001	2.87
F03	846.92 ± 14.05	0.106 ± 0.002	1.77
F04	733.88 ± 12.84	0.092 ± 0.002	3.23
F 05	694.87 ± 8.46	0.087 ± 0.001	4.18
Control	655.11 ± 8.34	0.082 ± 0.001	4.32

## *Ex-vivo* drug permeation studies

**Figure 24.**
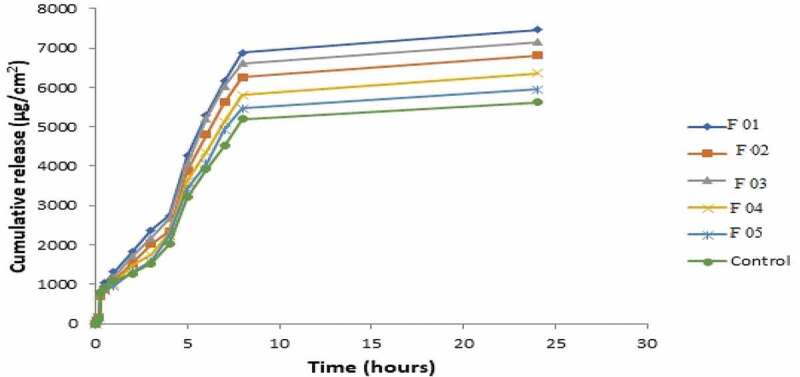
Permeation profiles of three NE formulations in comparison with control

**Figure 25.**
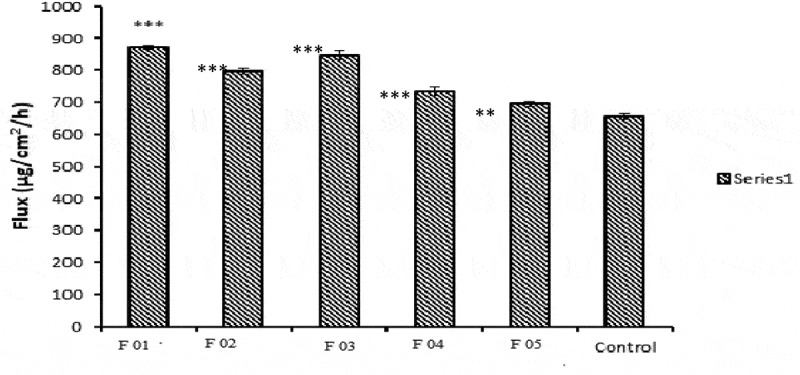
Permeation rate (flux) of three NE formulations in comparison with control. (*) represents significant difference of NE formulations flux from control

**Figure 26.**
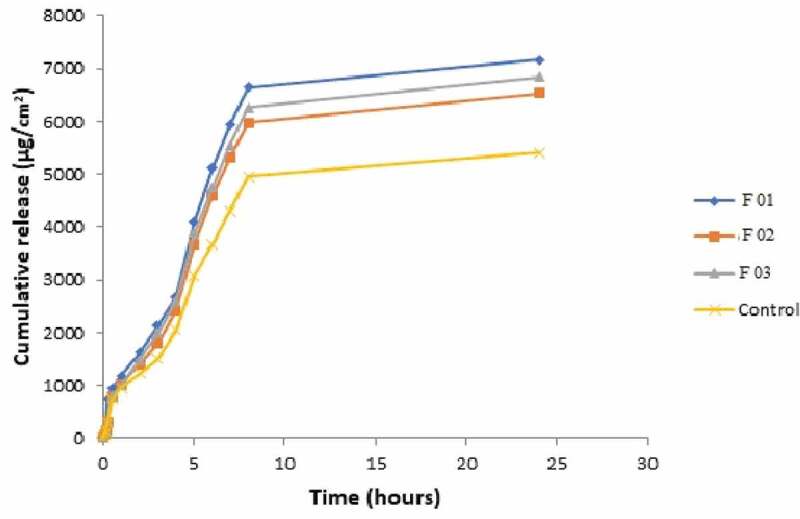
FTIR Spectrum of nanoemulsion (NE), miconazole and gelatin

For *ex-vivo* permeation studies, the formulations F01, F02, and F03 were selected from *in-vitro* data based on higher release rates. The release order was F01> F03> F02> Control as indicated in Figure 24. Formulation F01 showed maximum release profile, flux and lowest lag time because of smallest particle size and lowest viscosity. Thus it was considered as ideal formulation for further anti-fungal activity. There was significantly higher (*p* < 0.05) permeation rate values of all formulations in comparison to control which is simple solution of miconazole in ethanol as shown in Figure 25 and 26. Various permeation factors are given in Table 4.

**Table 4.** *Ex-vivo* permeation parameters of selected NE formulations

Nanoemulsion codes	Flux (µg/cm^2^/hr)	Permeability coefficient (cm/hr)	Lag time (min)
F01	842.07 ± 16.4	0.105 ± 0.002	2.05
F02	758.55 ± 13.99	0.095 ± 0.002	3.05
F03	791.1 ± 16.16	0.099 ± 0.002	1.96
Control	622.54 ± 14.95	0.078 ± 0.002	4.49

## Fourier transforms infra-red spectroscopy (FT-IR) of nanoemulsion

FTIR spectrum of pure gelatin displayed a broad peak at 3450 cm-1 and 3423 cm-1 which were due to stretching vibrations of secondary amine (-NH) as shown in Figure 25. Low intensity peaks at 2922 cm^−1^ and 2850 cm^−1^ can be attributed to C-H stretching. Strong peaks at 1680 cm^−1^ and 1640 cm^−1^ were associated to stretching due to carbonyl groups (C=O). Lastly, amine group (-NH) exhibited its bending between 1550 cm^−1^ and 1500 cm^−1^.

FTIR spectrum of pure miconazole showed characteristic peaks at 3180 cm-1 due to Imidazole ring structure (C-N stretching) as shown in Figure 25. Strong peak at 3107 cm^−1^ was presented by stretching of aromatic CH; broad peak at 2960 cm^−1^ was due to stretching vibrations of aliphatic CH2 group and peaks in the range of 1500 to 1600 cm^−1^ can be ascribed to stretching of aromatic C=C. Strong peaks at 1385 cm^−1^ and 1330 cm^−1^ revealed to C-H bending vibrations and C-N stretching vibrations, respectively. Additionally, peak in the range of 1000 to 1100 cm^−1^ corresponds to C-C stretching vibrations. The spectra of polymers and drug loaded NE have been shown in Figure 25.

FTIR spectrum of developed NE showed a broad valley in the range of 3600-3000 cm-1 which may be due to stretching vibrations of free –OH of gelatin, –NH of Amide groups of gelatin and amidazole group of miconazole. –OH stretching vibrations are overlapped by –NH stretching vibrations forming a broad valley. Characteristics peaks of gelatin and miconazole were present at 2922 cm^−1^ and 2850 cm^−1^ (C-H stretching). Similarly, bands were also observed at 1680 cm^−1^, 1640 cm^−1^, 1550 cm^−1^ and 1500 cm^−1^ (stretching due to carbonyl groups and amine group (-NH) bending vibrations); 1385 cm^−1^ (C-H bending vibrations); 1330 (C-N stretching vibrations) and 1100 cm^−1^ (C-C stretching vibrations). These characteristic bands of miconazole confirmed the entrapment of drug in the NE structure.

## Conclusion

NEs were successfully by high pressure homogenization techniques. NEs were prepared by varying the ratios of lecithin, gelatin, ethanol and water. Amount of miconazole remained same in all formulation. Total five formulations were formulated by varying the ratios of polymer, surfactant, oil and water phases. All the formulated showed good drug loading and consequenstly good drug release. NEs can be easily penetrate into the skin and can treat the fungal infections. There was an increase in the permeation of nanoels in skin due to their nanosize particlularly. Drug release was good in the basic pH buffer. NE have shown to inhibit and retard the growth of strain of *C.albicans* as predicted from in vivo and ex vivo studies. Ternary pseudo phase diagram was also constructed for the prepared NE using excel sheet graph. XRD of NEs have shown that they were amorphous in nature and its also helped the formulation to gain more stability. DSC results have predited absence of drug melting peak which indicates molecular dispersion of drug in the prepared NE. SEM analysis and zeta sizer have confirmed that prepared NE had size in the nano ranges and globule were evenly distibuted throughout the formulation. Stability studies of NE as observed for 90 days proved miconazole present in NE remained stable. At the end it was inferred that NE were stable as well as had good anti-fungal activity.
